# Silicon-Containing Polymeric Materials

**DOI:** 10.3390/polym13020188

**Published:** 2021-01-07

**Authors:** Ignazio Blanco

**Affiliations:** Department of Civil Engineering and Architecture and INSTM UdR, University of Catania, Viale Andrea Doria 6, 95125 Catania, Italy; iblanco@unict.it

When thinking about a chemical element that has contributed to the technological progress over the last two centuries, carbon and all carbon-based materials immediately come to mind. Particularly during the last century, Silicon and its related materials follow very closely. The versatility of silicon-based materials and silicon’s abundance in the earth’s crust ensure that it will continue to play, in the years to come, a vital role in everyday life. In addition to silicones or polysiloxanes [1,2,3,4], which have been known and manufactured for many years, silica-reinforced polymers, silsesquioxanes, and POSS-based polymers also offer a multitude of very useful consumer products [5]. The combination of silicon and oxygen atoms with organic groups has led to the generation of new and modified silicon-containing polymeric materials, thus providing an exciting mixture of properties and offering a wide spectrum of practical applications. This is why the editorial Board of Polymers decided to institute, belong to the section of Polymer Chemistry, the Topical Collection “Silicon-Containing Polymeric Materials”, offering the possibility of publishing novel researches exploring silicon-containing polymeric materials and their applications.

The Topical collection hosted three special issues: POSS-Based Polymers; Siloxane-Based Polymers; Silsesquioxane (POSS) Polymers, Copolymers and Nanoparticles; and it collected 112 papers and many views, downloads and citations, demonstrating the vitality of this area of the polymeric world. The highly cited works are summarized in this editorial. 

Catauro and co-workers used pure silica and silica-based hybrids materials, containing polyethylene glycol (PEG), to synthesize, using the sol-gel technique, novel phenol-based materials with phenolic antioxidant chlorogenic acid (CGA) at various percentages, aiming to reduce the matrix’s degradation and thus the loss of its properties. Thermal and soaking analyses demonstrated the improvement of matrix performance by maintaining its bioactivity, despite the presence of PEG and CGA [6].

Liang et al. used surface modified and unmodified fumed nano-silica for reinforcing silicone rubber composites for high voltage insulating materials in power transmission and substation systems. During the evaluation of silicone composites they observed modifications in the dielectric response with both of the two type of silica used: a paralleled combination of Maxwell-Wagner-Sillars interface polarization and DC conduction by using the modified silica; a quasi-DC transport process by using the unmodified nano-silica. They also observed an improvement in the mechanical strength with increasing silica content [7].

Elías-Zúñiga and colleagues studied magnetorheological elastomers (MREs) based on a silicone–rubber matrix and carbonyl iron microparticles. After the preparation of the samples, with and without the application of an external magnetic field, the researchers evaluated the rheological behavior of isotropic and anisotropic composites, finally plotting the Cole-Cole diagrams to quantify the interfacial adhesion between particles and silicone. They recorded a different performance for the anisotropic and isotropic composites, with the latter showing a reduction in the loss and storage modulus compared with the bare material. Conversely, in the anisotropic composites, the good particles’ dispersion led to an increase of the modulus and damping properties and an increase of the loss and the storage modulus compared to those of the bare material [8].

Marine antifouling coatings based on silicone elastomers, unlike biocide-based ones, do not release biocides into the marine environment, but the antifouling efficacy during idle periods is strongly reduced. With the aim to enhance this specific performance, Gevaux et al. reinforced a silicone matrix with various amounts of hydrolysable polymers, such as poly(ε-caprolactone) (PCL), poly(bis(trimethylsilyloxy)methylsilyl methacrylate). The France researchers evaluated the attachment of macrofoulers on these coatings by field tests in the Mediterranean Sea, demonstrating the short or long-term antifouling effect of these hydrolyzable polymers embedded in the silicone matrix [9]. 

Lopez-Manchado and co-workers applied themselves in the tailoring of the mechanical and transport properties of silicone rubber nanocomposites with thermally reduced Graphene Oxide (TRGO), aiming to improve their thermal conductivity for application in power electronics and electric motors. The researchers from Spain, by regulating the morphological and structural characteristics of TRGO, as well as its concentration in the composite, demonstrated that TRGOs with high specific surface area and a partial aromatic restoration are suitable to improve both the mechanical properties and the electrical conductivity of silicone rubber composites [10]. 

Yang et al. employed their studies on the balance of the properties requirement of silicone-based hydrogel contact lenses, such as water content, oxygen permeability, optical transparency, contact angle, protein adsorption and cell toxicity. They observed that the presence of 3-(methacryloyloxy)propyltris(trimethylsiloxy)silane enhances the oxygen permeability while the presence of N,N-dimethylacrylamide and 1-vinyl-2-pyrrolidinone influenced the hydrophilicity of the hydrogels. Since their hydrogel showed good oxygen permeability, stiffness and optical transparency, as well as anti-protein adsorption, they concluded that this silicone-hydrogel can be commercialized for contact lens [11].

Instead of the random and more commercialized cage-structured silsesquioxanes, Bae et al. designed and synthesized, in a very short time by sol–gel method and free-radical polymerization, a structurally ordered siloxane backbone with a ladder-like structure. This structure turned out an essential factor for high performance, they tested by optical, thermal and mechanical characterization, suggesting the ladder-like structured methacrylate siloxane hybrid as a powerful alternative for the methacrylate polysilsesquioxane [12].

Niemczyk et al., by the means of Thermogravimetry-Fourier Transform Infrared Spectroscopy (TG-FTIR) and cone calorimeter analysis, evaluated the effects of a novel group of silsesquioxanes, namely siloxane-silsesquioxane resins (S4SQ), as possible flame retardants for polypropylene (PP). By using a small amount of S4SQ, they observed a beneficial effect due to the formation of a continuous ceramic layer on the material surface during its combustion, which improved both thermal stability and flame retardancy of the PP. Based on the TG-FTIR results the polish researchers proposed a possible mechanism for the degradation of S4SQ resins to explain their role during the combustion of the PP/S4SQ composites [13].

Tong and his colleagues focused their studies on the effects of the variation in electron-deficient units on the properties of photovoltaic polymers. Namely, they designed and synthesized three alternated D- p-A type 5,10-bis(triisopropylsilylethynyl)dithieno[2,3-*d*:2′,3′-*d*’]-benzo[1,2-*b*:4,5-*b*’]dithiophene (DTBDT-TIPS)-based semiconducting conjugated copolymers (CPs), PDTBDT-TIPS-DTBT-OD, PDTBDT-TIPS-DTFBT-OD, and PDTBDT-TIPS-DTNT-OD, bearing different A units, including benzothiadiazole (BT), 5,6-difluorinated-BT (FBT) and naphtho[1,2-*c*:5,6-*c*’]-bis[1,2,5]thiadiazole (NT). Their investigation, and the obtained results, certified the replacement of BT with NT in a D- p-A type polymer backbone as a good strategy for tuning the molecular structure aiming at producing highly efficient polymer solar cells (PSCs). In particular, the optimized photovoltaic device based on the PDTBDT-TIPS-DTNT-OD showed a drastic enhancement of the power conversion efficiency, and deteriorated after incorporating fluorine into the BT, due to the oversized aggregation and large phase separation morphology in the blend films [14].

Łagód and co-workers studied the effects of polysiloxanes hydrophobised’s incorporation in basalt fibres–reinforced cement mortars. Specifically, they evaluated the roughness, the adhesion properties and the surface free energy (SFE), finding a decrease in the surface roughness and an increase in the frost resistance. Unfortunately, the polymer admixture led to the formation of chemical bonds among the concrete components, influencing the changes in the microstructure and the strength parameters. In particular, the hardening process of polysiloxane gel in the aqueous environment of cement slurry and cement hydration in the presence of polymer compound are disrupted, and this is the reason why the researchers do not recommend the application of polysiloxanes in this type of mortars [15].

A completely different application area of polysiloxanes was highlighted in the review reported in [16], where the employment of these silicon-based materials in the field of controlled drug delivery and theranostics was analyzed. Biocompatibility, versatility, physical and chemical resistance and the ability to be functionalized of these materials offer the opportunity to overcome one of the historical problems of medicine, namely of not interfacing at the best of diagnosis and therapy. In this review the research progress in recent years is shown, which offered the opportunity to build a platform able to ferry drugs, and load onto them both imaging and therapeutic functions, thus creating nanosystems capable of diagnosis, drug delivery and monitoring of therapeutic response.

Despite the fact that methyl-terminated poly(dimethylsiloxanes) (PDMSs) are not suitable for surface modification due to the absence of readily hydrolyzable groups, Le et al. proposed a new strategy for surface modification based on the use of silica nanoparticles, poly(dimethylsiloxanes) and diethyl carbonate (DEC) as initiator. They carried out a spectroscopic and rheologic characterization, finding the full involvement of free silanol groups in the chemisorption process, hydrophobic surface properties and thixotropic behavior in industrial oil for the PDMS-x/DEC mixture [17].

In recent years, three-dimensional PDMS foams have attracted increased attention for use as a scaffold for different decorating agents but they contain residual unreacted low molecular weight species that need to be removed in order to obtain a standard and chemically stable material. Duce and co-workers proposed a cleaning procedure for PDMS foams using a sugar templating process with the employment of two solvents (hexane and ethanol) as cleaning agents. Then they studied the PDMS’s decomposition, before and after the cleaning procedure, comparing the obtained data with those of a PDMS bulk used as a reference. In particular, the Italian researchers highlighted the importance of thermogravimetric analysis (TGA) in confirming the removal by the washing of low molecular weight oligomers and unreacted reagents, information not obtainable from spectroscopic or morphological studies [18].

Since the development of porous polymeric membranes represents an important area of application in separation technology, Tan and Rodrigue proposed a review work to summarize the studies on PDMS development from the perspectives of membrane production. They reviewed six methods for membrane fabrication, such as thermally induced phase separation, melt-spinning and cold-stretching, phase separation micromolding, imprinting/soft molding, manual punching and three-dimensional printing [19].

Probably the hottest topic of this collection is represented by polyhedral oligomeric silsesquioxanes (POSSs). The wide employment of these materials in the last 25 years (it is said of their rebirth) is attributable to the setup of a new and easy method of synthesis by Feher [20,21] and to the intuition of Lichtenhan about the potential applications of POSSs in the polymer’s sector for making hybrid composites [22,23]. POSSs are composed of a silicon and oxygen cage, which is externally completed by organic groups that are covalently bonded with the silicon atoms. The most common value of *n* is 8, thus generating a very highly symmetric structure, sometimes indicated in the literature with the symbol T_8_, having a diameter usually ranging in 1.5–3 nm [24]. In comparison with the other commonly used fillers, POSSs possess the advantage of being molecules, thus they offer various possibilities of tuning their organic periphery as a function of the matrix which host them, thus ensuring better compatibility.

Zhang et al. modified the organic groups obtaining acrylolsobutyl polyhedral oligomeric silsesquioxane (MAPOSS) to be used in reinforcing, via radical polymerization. Poly(N-isopropylacrylamide) (PNIPAM) was used as drug carrier for 5-fluorouracil. They observed how the introduction of MAPOSS into the polymer network led to a strong enhancement of the compressive modulus of the hydrogel and how by setting the MAPOSS/ polyethylene glycol (pore-forming agent) ratio it was possible to obtain a good compromise between the mechanical properties and swelling behavior, thus achieving stable and sustained drug release [25].

Liu and colleagues prepared resin-based composites by inserting in the POSS’s corona methacryl groups and then adding different percentages of methacryl POSS to the matrix by light curing method. The Chinese researchers then carried out a wide investigation to evaluate the compatibility, double bond conversion, volumetric shrinkage, hardness, modulus and resistance of the dental resins, which showed uniform dispersion and good compatibility of MAPOSS with the matrix [26].

Exploiting the possibility offered by POSS to create Hybrid materials, Wei Kuo and Gamal Mohamed discussed the various methods for inserting these nanoparticles into polyimide (PI) matrix, a very important techno-polymer. Among the different methods of preparation they evaluate the covalent chemical bonding and physical blending, as well as the influence of the POSS units on the physical properties of the PIs [27].

The work of Dai and co-workers always falls in the framework of the hybrids, which synthesized a novel copolymer by using glycidyl methacrylate (GMA), bis-9,10-dihydro-9-oxa-10-phosphaphenanthrene-10-oxide methacrylate (bisDOPOMA), functionalized graphene oxide (GO) and methacryloisobutyl POSS (POSSMA) for flame retardant purpose. They observed how the adding of 4 wt% of the hybrid reinforcement increases the mechanical strength, thermal properties and the limiting oxygen index (LOI) of the epoxy resin [28].

Członka et al. worked on improving the thermo-mechanical performance of rigid polyurethane foams (RPUFs) by using different percentage (0.5–5 wt%) of closed-cage nanostructure, such as aminopropyl isobutyl-POSS (APIB-POSS) and aminoethylaminopropylisobutyl-POSS (AEAPIB-POSS). The morphology, viscoelastic, mechanical and thermal behavior together with thermal conductivity and application properties were evaluated, showing the best results when APIB-POSS was added to the RPUFs matrix at 0.5 wt% [29].

Other Polish researchers, Dudziec and co-workers, provided an exhaustive review regarding the application of organic optoelectronic materials, indicating the possibility of their use in the construction of organic–inorganic hybrid materials by using the organic POSSs’ corona architecture linked to their rigid silica cores. They reviewed a significant number of works dealing with the preparation of POSS-based organic optoelectronic as well as photoluminescent (PL) materials, focusing on synthesis and catalytic methods to prepare silsesquioxane systems for photoactive materials [30].

Remaining in the field of optoelectronic devices, Wei et al. synthesized and characterized a series of optically transparent shape memory polyimide films with various POSS contents. The use of the inorganic POSS structure gave to the hybrid films excellent thermal stability, with a considerable increase in the glass transition temperature (*T*_g_) value (+20 °C), showing the successfully use of POSS in developing transparent polyimides with excellent thermal stability and shape memory effect for space applications [31].

Dong et al. designed and fabricated raspberry-like hollow-spheres-on-sphere (HSOS) polysilsesquioxane (PSQ) particles with reactive surfaces, uniform sizes and monodisperse properties were rational to immobilize gold nanoparticles for the catalytic reduction of 4-nitrophenol. The synthesis process was monitored and the formation mechanism of the hierarchical particles was investigated in detail by the time of the study through imaging the particles at regular time intervals during the reaction. The prepared material has shown potential applications in separations, drug delivery and heterogeneous catalysis [32].
